# Supplementary material for: Antiferromagnetism and superconductivity in the half-Heusler semimetal HoPdBi
Source: Sci Rep. 2016 Jan 5;6:18797. doi: 10.1038/srep18797 (PMC4700465; doi:10.1038/srep18797)
Supplement: Supplementary Information [file srep18797-s1.pdf]

SUPPLEMENTARY MATERIAL

Antiferromagnetism and superconductivity in the half-Heusler semimetal HoPdBi

O. Pavlosiuk, D. Kaczorowski, X. Fabreges, A. Gukasov and P. Wiśniewski*

* Correspondence to: p.wisniewski@int.pan.wroc.pl

MATERIAL CHARACTERIZATION

Energy-dispersive X-ray spectroscopy

Examples of the EDS spectrum and the SEM image obtained for the single crystals of HoPdBi are presented in Fig. S1. The chemical composition derived from EDS analysis is $Ho_{31.3(3)}Pd_{35.8(4)}Bi_{32.9(4)}$, in a fairly good accord with the ideal equiatomic one. The crystals were found homogeneous and no foreign phase was detected.

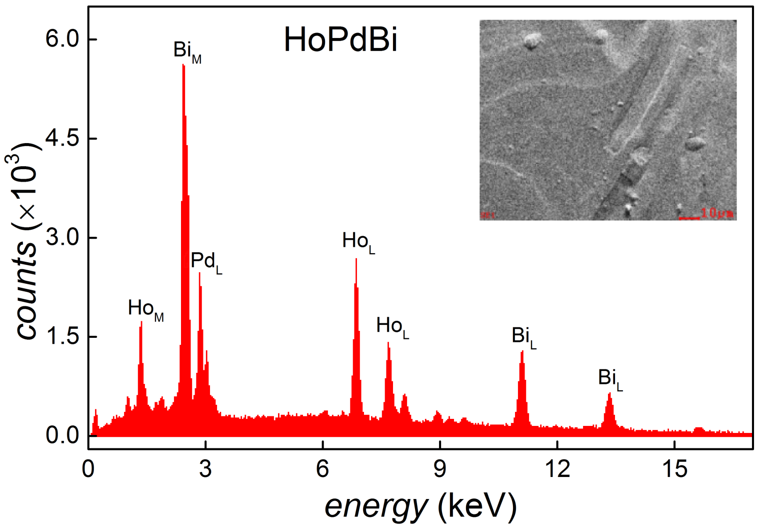

Figure S1. EDS spectrum and SEM image for single-crystalline HoPdBi.

X-ray diffraction

The PXRD results (see Fig. S2) confirmed a single-phase character of the obtained single crystals of HoPdBi. The X-ray diffraction pattern can be fully indexed within the $F\bar{4}3m$ space group, characteristic of half-Heusler compounds, and yields the lattice parameter $a = 6.613(2)$ Å.

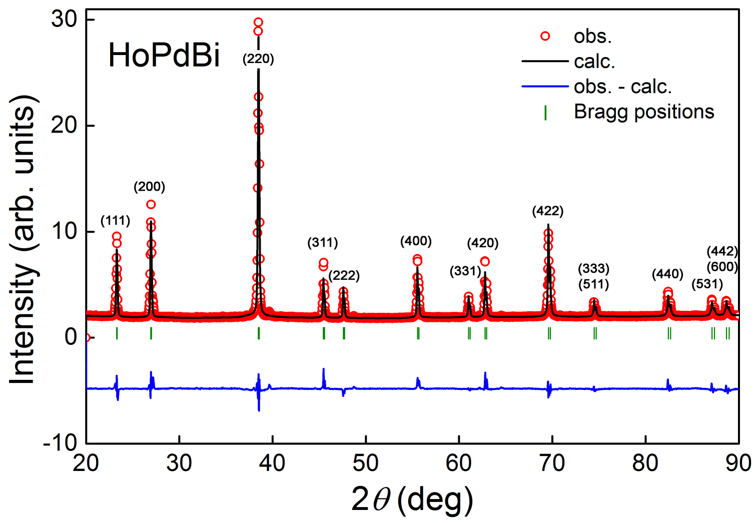

Figure S2. XRD diffractogram for powdered single crystals of HoPdBi.

This value is in perfect accord with the literature value $6.610(1)$ Å determined for polycrystalline sample,[1] and the value 6.605 Å reported recently for for powdered single crystals.[2] As can be inferred from Fig. S2, the experimental PXRD pattern of our single-crystalline HoPdBi can be very well modeled with the MgAgAs-type crystal structure with the Ho atoms located at the crystallographic 4a $(0, 0, 0)$ sites, the Pd atoms occupying the 4c $(1/4, 1/4, 1/4)$ sites, and the Bi atoms placed at the 4b $(1/2, 1/2, 1/2)$ sites.

Analysis of the negative magnetoresistance

Following the analysis applied for HoNiSb in Ref. 3 we fitted our $\rho(B)$ data with the de Gennes-Friedel function: $\rho(B) = \rho_0[1 - (\beta(CB/(T - \theta_{\mathrm{CW}})))^2]$, where $C = N\mu^2/k_{\mathrm{B}}$ is Curie constant and θ_{CW} is paramagnetic Curie-Weiss temperature and β denotes Brillouin function. Fitting data collected at 2.5 K, using θ_{CW} fixed at -9.4 K detrmined in Ref. 4, gives a magnetic moment $\mu = 5.96\mu_{\mathrm{B}}$ for Ho, close to 6.9 μ_{B} obtained by magnetization measurement but lower than the theoretical value for a free Ho ion. This reduction may be justified by crystal field effects. That fit is shown in Fig. S3, together with those performed for data collected at higher temperatures (yielding similar values of μ).

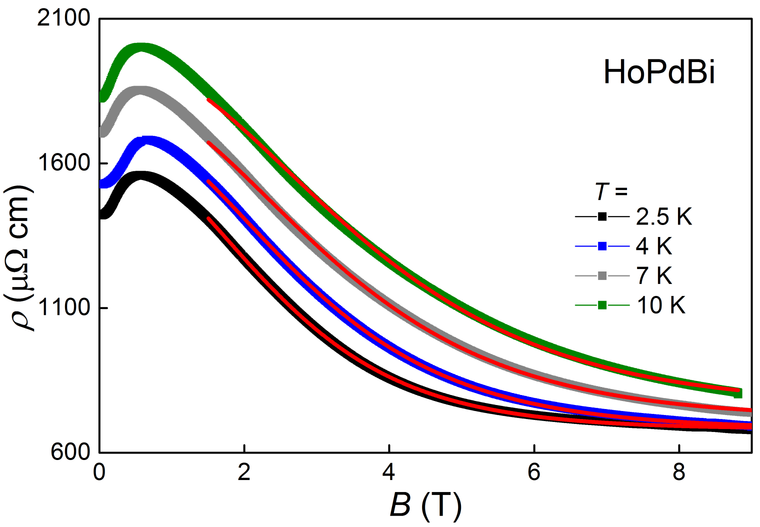

Figure S3. Field dependence of the resistivity of HoPdBi at different temperatures. Red lines represent de Gennes-Friedel function fitted to experimental data.

REFERENCES

[1]Marazza, R., Rossi, D. & Ferro, R. MgAgAs-type phases in the ternary systems of rare-earths with palladium and bismuth. *Gazz. Chim. Ital.* **110**, 357 (1980).

[2]Nikitin, A. M. *et al.* Magnetic and superconducting phase diagram of the half-Heusler topological semimetal HoPdBi. *J. Phys. Condens. Matter* **27**, 275701 (2015).

[3]Karla, I., Pierre, J., & Skolozdra, R. Physical properties and giant magnetoresistance in RNiSb compounds. *J. Alloys Compds.* **265**, 42 (1998).

[4]Nakajima, Y. *et al.* Topological RPdBi half-Heusler semimetals: A new family of noncentrosymmetric magnetic superconductors. *Sci. Adv.* **1**, e1500242 (2015).
